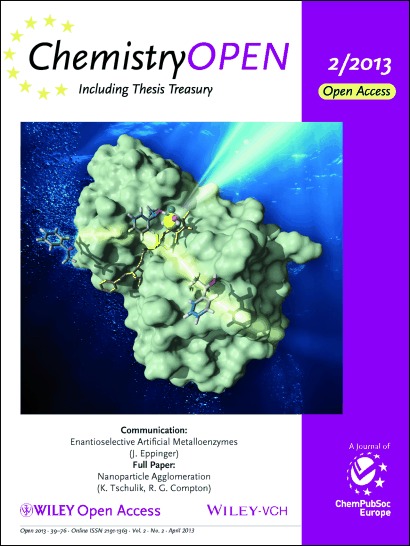# Metal-Conjugated Affinity Labels: A New Concept to Create Enantioselective Artificial Metalloenzymes

**DOI:** 10.1002/open.201300015

**Published:** 2013-04-23

## Abstract

Invited for this month′s cover is the group of Prof. Jörg Eppinger. The cover picture illustrates the concept of using metal-conjugated affinity labels (m-ALs) to convert proteases into well-defined and catalytically active artificial metalloenzymes. For more details, see the Communication on p. 50 ff.


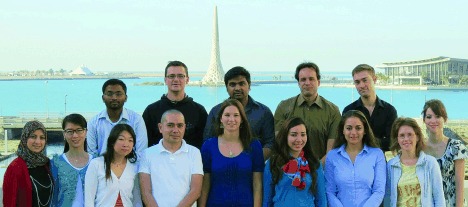


The Group of Jörg Eppinger


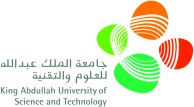


Division of Physical Sciences and Engineering and KAUST Catalysis Center, KCC King Abdullah University of Science and Technology, KAUST Thuwal 23955-6900 (Saudi Arabia) E-mail: jorg.eppinger@kaust.edu.sa

## What prompted you to investigate this topic/problem?

Studies on artificial metalloenzymes have developed into a vibrant area of research. It is expected that artificial metalloenzymes will be able to combine the best of enzymatic and homogenous catalysis, that is, broad catalytic scope, high selectivity and activity under mild, aqueous conditions. Recently, Ward et al. were able to show that supramolecular anchoring of a biotin-conjugated metal center on streptavidin followed by directed evolution of the protein host can lead to highly enatioselective and active organometallic enzyme hybrid (OMEH) catalysts. Unfortunately, application of this very successful approach is limited by the properties of the ligand protein and the stability of the supramolecular interaction. Therefore, our aim was to develop a modular platform, where a variety of proteins can be combined with diverse artificial cofactors. The OMEH catalyst’s selectivity, stability and solubility will then be tuned by simple testing of protein/cofactor combinations to identify lead candidates.

## What is the most significant result of this study?

Our work presents a novel method to generate well-defined and catalytically active artificial metalloenzymes. Covalent attachment of artificial cofactors was applied previously to convert proteases into organometallic enzyme hybrids. However, enantioselectivities reached by such covalent approaches stayed well behind those of Ward’s biotin–streptavidin conjugates. We reasoned that covalent approaches so far suffered from an ill-defined orientation of the metal center on the protein surface. Using specifically designed metal-conjugated affinity labels to introduce metal centers into the binding pocket of cysteine proteases, we generate a structurally distinct binding mode of the cofactor. Our data clearly show the importance of an affinity tail to determine the orientation of a cofactor-conjugated metal center and the influence of the protein host on the enantiomeric ratios achieved.

## What other topics are you working on at the moment?

The interface of chemistry and biology holds solutions to many current problems. We consider the combination of molecular processes of living cells with the tools and principles of chemistry a particularly fruitful approach to establish novel types of catalysts. Therefore we are interested in: (i) development of (green chemistry) aqueous organometallic catalysis protocols for cross-coupling, hydrogenation, pericyclic and polymerization reactions; (ii) development of methods to incorporate metal centers into proteins (e.g., as presented in this article); (iii) investigation of the interaction of metal centers with proteins and peptides, as well as discovery and characterization of metalloproteins; and (iv) applications of bio-electrochemical conversions involving H_2_ or CO_2_. The unique location of our laboratory at the shores of the Red Sea, expertise of collaborators from KAUST Marine Research Center and support by KAUST’s core-facilities give us direct access to data from novel extremophiles, which directly feed into our research projects.